# Endovascular Treatment and Peri-interventional Management of Ruptured Cerebrovascular Lesions During Pregnancy: Case Series and Case-based Systematic Review

**DOI:** 10.1007/s00062-023-01287-x

**Published:** 2023-05-31

**Authors:** Manina M. Etter, Anh Nguyen, Alex Brehm, Christoph Aberle, Ioannis Tsogkas, Raphael Guzman, Adam A. Dmytriw, Carmen Parra-Farinas, Justin R. Mascitelli, Vitor Mendes Pereira, Robert M. Starke, Isabel Fragata, João Reis, Stacey Quintero Wolfe, Guilherme B. Porto, Alejandro M. Spiotta, Marios-Nikos Psychogios

**Affiliations:** 1 Department of Neuroradiology, Clinic of Radiology and Nuclear Medicine, University Hospital Basel, Petersgraben 4, 4031 Basel, Switzerland; 2 Department of Radiology, Clinic of Radiology and Nuclear Medicine, University Hospital Basel, Basel, Switzerland; 3 Department of Neurosurgery, University Hospital Basel, Basel, Switzerland; 4 Neurointerventional Program, Departments of Medical Imaging & Clinical Neurological Sciences, London Health Sciences Centre, Western University, London, UK; 5 Division of Diagnostic and Therapeutic Neuroradiology, Department of Medical Imaging, St. Michael’s Hospital, University of Toronto, Toronto, Canada; 6 Department of Neurosurgery, University of Texas Health Science Center at San Antonio, San Antonio, TX USA; 7 Jackson Health System, Lois Pope Life Center, Department of Neurological Surgery, University of Miami, Miami, FL USA; 8 Department of Neuroradiology, Centro Hospitalar Lisboa Central, Lisbon, Portugal; 9 Departments of Neurological Surgery and Radiology, Wake Forest, School of Medicine, Winston-Salem, NC USA; 10 Department of Neurosurgery, Medical University of South Carolina, Charleston, SC USA

**Keywords:** Pregnancy, Cerebrovascular malformation, Cerebral aneurysm, Cerebrovascular arteriovenous malformation, Endovascular treatment

## Abstract

**Purpose:**

Hemorrhagic stroke, particularly occurring from ruptured cerebrovascular malformations, is responsible for 5–12% of all maternal deaths during pregnancy and the puerperium. Whether endovascular treatment is feasible and safe for both the mother and the fetus, is still a matter of debate. The main objective of this case series and systematic review was to share our multi-institutional experience and to assess the feasibility and safety of endovascular treatment during pregnancy, as well as the corresponding maternal and fetal outcomes based on currently available evidence.

**Methods:**

We report a case series of 12 pregnant women presenting with hemorrhagic stroke from ruptured cerebrovascular arteriovenous malformations or aneurysms who underwent endovascular treatment prior to delivery. A systematic literature review of pregnant patients with endovascular treated cerebrovascular malformations, published between 1995 and 2022, was performed. Clinical patient information, detailed treatment strategies, maternal and fetal outcomes as well as information on the delivery were collected and assessed.

**Results:**

In most patients the course was uneventful and an excellent outcome without significant neurological deficits (mRS ≤ 1) was achieved. Furthermore, the maternal outcome was not worse compared to the general population who underwent endovascular treatment of ruptured vascular brain lesions. Also, in most cases a healthy fetus was born.

**Conclusion:**

Endovascular treatment of ruptured cerebrovascular malformations during pregnancy is safe and feasible regarding both aspects, the maternal and fetal outcomes. Still, a stronger knowledge base is needed to correctly approach future cases of intracranial hemorrhage in the pregnant population.

**Supplementary Information:**

The online version of this article (10.1007/s00062-023-01287-x) contains supplementary material, which is available to authorized users.

## Introduction

Hemorrhagic stroke is the underlying cause in 5–12% of all maternal deaths during pregnancy and the puerperium. The third most common non-obstetric cause of maternal mortality is caused by aneurysmal subarachnoid hemorrhage. In addition, hemorrhagic stroke accounts for up to 60% of all strokes arising during pregnancy [1–3]. The literature so far suggests that the period of pregnancy, particularly the third trimester and 12 weeks postpartum, is associated with an increased risk of hemorrhagic stroke, mainly occurring from ruptured cerebral aneurysms and arteriovenous malformations (AVMs) [1, 4]; however, it is still a matter of debate if causality between pregnancy and the formation and/or rupture of cerebral malformations exists. Either way, the management of hemorrhagic stroke during pregnancy raises serious concerns regarding the maternal and fetal outcomes, due to lacking guidelines, limited data and poor understanding of the disease’s natural history in pregnancy.

Maternal and fetal wellness are intertwined, and risks of premature birth may conflict with maternal stroke management. Diagnostic imaging studies and treatment options are limited due to possible radiation, pharmacological as well as surgical side effects to the fetus and mother. For instance, direct open surgery carries inherent risks, such as intraoperative hemorrhage which in turn could impair the placental and uterine circulations, possibly threatening the fetus [5], while endovascular approaches carry the concern of radiation exposure. Endovascular approaches, however, have been increasingly considered as a treatment option in pregnancy [5, 6]. Recent improvements of the used devices and different procedural techniques, such as radial approaches, allow minimization of the possible maternal and fetal risks and therefore encourage the trend of endovascular treatment in pregnant women [5, 7].

The main objective of this case series and systematic review was to share our multi-institutional experience and to assess the feasibility of endovascular treatment during pregnancy, as well as the corresponding maternal and fetal outcomes based on available evidence.

## Methods

### Patient Population of the Case Series

Patient histories of pregnant women with ruptured and treated brain aneurysms and AVMs were retrospectively collected using a multi-institutional network of interventional neuroradiologists and neurosurgeons. We did not obtain an additional ethical permit as a general research consent for the use of retrospective and anonymized data was obtained for each hospitalized patient.

### Review of the Literature

**Fig. 1:**
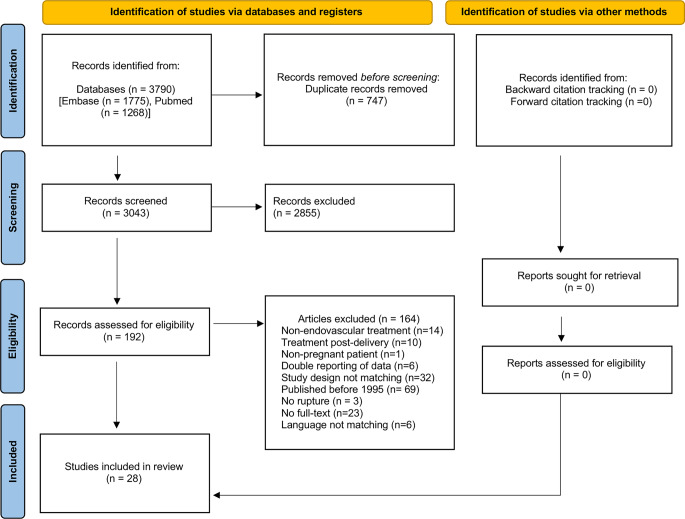
Flow-chart of included and excluded articles, following the Preferred Reporting Items for Systematic Reviews and Meta-analyses (PRISMA) guidelines

A systematic literature search of PubMed and Embase was performed to identify studies assessing the management of cerebrovascular malformations in pregnancy (Fig. 1). There were no restrictions on the type of study except for literature reviews. Search strings around the concepts hemorrhagic stroke, pregnancy and cerebrovascular malformations were composed of subject headings and text work synonyms. The complete search strings are deposited in the Online Resources file (date of last search 20 July 2022). Results published from January 1995 until July 2022 were independently assessed by two reviewers. After removal of duplicates, the titles and abstracts were reviewed and a list of suitable studies was generated. Full-text articles were only obtained if the abstracts were considered eligible by both independent reviewers. The remaining studies underwent a full text evaluation to compile a final list of references. According to Preferred Reporting Items for Systematic Reviews and Meta-analyses (PRISMA), we additionally screened the reference lists of the selected articles. All discrepancies and disagreements of the review were addressed by input from a third and fourth reviewer in all steps of the review. English and German language restrictions were applied.

### Studies Selection Criteria and Patient Population

We determined the research question and patient population using the Patient, Intervention, Comparison and Outcome (PICO) strategy. Our patient population included pregnant females with ruptured cerebral AVMs and aneurysms, who underwent endovascular treatment prior to delivery. The outcomes of interest were maternal and fetal mortality, maternal ischemic events, technical success rates, maternal and fetal clinical outcomes and pregnancy outcome. We did not include a control population. Non-randomized prospective cohort studies, as well as retrospective cohort studies, case series and case reports were included. Randomized controlled trials were not available for this research question.

### Statistical Analysis

Statistical analysis was performed using GraphPad Prism 9 (GraphPad Software, San Diego, CA, USA, https://www.graphpad.com/, 2021). Parametric variables were assessed for normality. If they were normally distributed, they were stated as mean ± standard deviation (SD). Non-normally distributed parametric variables and non-parametric or ordinary variables are presented as median and interquartile range (IQR). No interference statistics were performed.

## Results

### Case Series

An overview of the patients’ characteristics is depicted in Table 1. A total of 12 patients were included in our case series. The average age was 31 ± 7 years. Pre-existing arterial hypertension was present in three patients and two had a positive family history of cerebral aneurysms. One of the patients developed pregnancy-related diabetes mellitus and one suffered from preeclampsia.

**Table 1:** Characteristic of all patients included in the case series

No. of patients	12
Age (years, mean, SD)	31 ± 7
Hypertonic, *n* (%)	3 (25%)
Pregnancy-related diabetes mellitus	1 (8.3%)
Pre-eclampsia	1 (8.3%)
Family history	2 (16.6%; both aneurysms)
Known aneurysm or AVM ^a^	0 (0%)
Prior antiplatelet treatment	1 (8.3%)
Prior heparin treatment	0 (0%)
Premorbid mRS ^b^ > 1	1 (8.3%)
Days from symptom onset to diagnosis > 2	2 (16.6%)
Headache	12 (100%)
Focal deficit	5 (42%)
Coma	1 (8.3%)
NIHSS ^c^ at admission (done in 5 patients; median, range)	1 (0–12)
GCS ^d^ at admission (done in 11 patients; median, range)	15 (8–15)
Ruptured aneurysm, *n* (%)	7 (58.3%)
Ruptured AVM, *n* (%)	5 (41.7%)
mRS 0–2 of mother at 90 days (or discharge)	9 (75%)
Fetal outcome: healthy	11 (91.7%)

^a^ *AVM* arteriovenous malformation

^b^ *mRS* modified Rankin scale

^c^ *NIHSS* National Institutes of Health Stroke Scale

^d^ *GCS* Glasgow coma scale

In our case series, seven patients sustained intracranial hemorrhage due to a ruptured cerebral aneurysm. Aneurysm location was the posterior communicating artery in four cases, internal carotid artery (ICA), basilar artery and inferior posterior cerebellar artery in one case each. Regarding the aneurysm size, in three patient the length and width of the aneurysm was wider than 5 mm, whereas only one patient presented with an aneurysm width and length exceeding 7 mm. The Hunt and Hess grade has been reported in six patients and was I in three cases (50%), II in two cases (33.3%) and IV in one case (16.6%).

In five patients a ruptured AVM was the underlying cause of intracranial hemorrhage.

With respect to imaging characteristics, most AVMs were Spetzler-Martin (SM) grade 2 (*n* = 4, 66.6%), with one SM grade 3 and one SM grade 1. In three cases (60%) the AVM was located in an eloquent brain area, deep venous drainage was present in one case (20%) and two AVMs (40%) depicted an intranidal aneurysm.

The clinical presentation began with headache in 12 patients (100%), a focal neurological deficit in 5 patients (42%) and 1 patient developed coma (8%). Median National Institutes of Health Stroke Scale (NIHSS) at admission was 1 (range 0–12) and median Glasgow coma scale (GCS) was 15 (range 8–15). A maternal modified Ranking Scale (mRS) between 0 and 2 at 90 days or discharge was achieved in 9 patients (75%) and 3 patients (25%) had an mRS of 3 or higher.

The type of delivery was reported in 10 cases: 4 had a vaginal delivery (40%), caesarean section (CS) was performed in 5 cases (50%, all of them at full term) and 1 patient (10%) had not delivered at the time of data acquisition.

In 11 cases a healthy fetus was born, whereas 1 fetus suffered intracranial hemorrhage and ventriculomegaly after the mother underwent stent-assisted embolization of an ICA aneurysm and developed in-stent thrombosis with infarction in the right middle cerebral artery (MCA) territory, which necessitated decompressive hemicraniectomy. The maternal outcome resulted in an mRS of 3 and the fetus was naturally delivered.

**Fig. 2:**
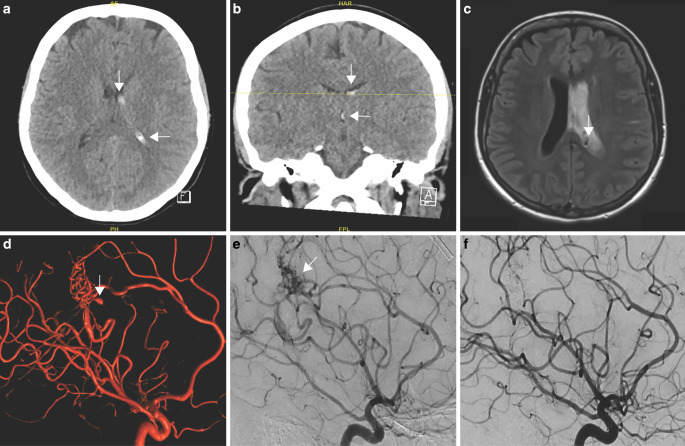
Illustrative case of a patient at 24 weeks of gestation. Axial (**a**) and coronal (**b**) cranial computed tomography (CT) at admission revealed intraventricular hemorrhage in the left lateral ventricle and in the third ventricle (*white arrow*s). **c** Cranial fluid-attenuated inversion recovery (FLAIR) magnetic resonance imaging (MRI) obtained at day 3 of hospitalization and after clinical worsening of the patient demonstrated a rebleeding in the left lateral ventricle and a cerebral arteriovenous malformation (AVM) (*white arrow*), representing the underlying cause of the hemorrhage. **d** Preinterventional three-dimensional volume-rendering technique (VRT) of the AVM with a related intranidal aneurysm (*white arrow*). **e** Preinterventional cerebral angiogram depicting the AVM (*white arrow*). **f** Postinterventional cerebral angiogram illustrating complete occlusion of the AVM embolized with Onyx

Preinterventional imaging and the angiographic outcome of an illustrative case is depicted in Fig. 2**.** The angiographic outcome was reported in 12 cases. In these five patients of our case series presenting with a ruptured AVM, full obliteration was achieved in three of five cases (60%) and in two patients (40%) postembolization resection was necessary as only partial embolization was performed. Embolization was performed using Onyx (Medtronic, Irvine, CA, USA) in three cases (60%) and N‑butyl cyanoacrylate (Histoacryl, B. Braun, Melsungen, Germany) glue in two (40%) cases.

Out of these seven patients of our case series presenting with ruptured cerebral aneurysms, in four cases (57%) full obliteration (Raymond and Roy I) was achieved and in three cases (43%) Raymond and Roy II with a residual aneurysm neck. Aneurysm treatment techniques were reported in six cases and consisted of aneurysm coil embolization in three cases (50%), stent-assisted coil embolization in two cases (33.3%) and balloon-assisted coil embolization in one case (16.6%).

In the subpopulation of patients diagnosed with ruptured AVMs, no periprocedural complications were reported. Periprocedural complications of aneurysm treatment were reported in three cases (43%). As previously described, one patient needed decompressive hemicraniectomy after in-stent thrombosis with subsequent infarction in the right MCA territory. One patient suffered a rerupture of the initially coiled aneurysm and in another patient a coil extruded through the aneurysm dome with transient rebleeding during the coiling procedure.

### Literature Review

After removal of duplicate records, our literature search identified 3043 potentially relevant and unique articles out of which 192 were subsequently assessed for eligibility. In total, we identified 28 matching studies comprising 37 patients for further data extraction (Fig. 1). All of the included studies were retrospective cohort studies, case series and case reports. Some of them contained an additional review of the literature.

A total of 37 patients were included in this study. Detailed patient characteristics are depicted in Table 2**. **The percentage refers to the total of included patients (*n* = 37). Regarding the fetal outcome the percentage in the manuscript text refers to a total number of 36. The mean age was 27.4 ± 6 years and the median gestational week at presentation was 21 weeks. The main symptom at presentation was headache, which was present in 27 patients (73%). Initial cranial imaging demonstrated intracerebral hemorrhage in 14 (38%), subarachnoid hemorrhage in 21 (57%) and intraventricular hemorrhage in 7 (19%) cases. All patients were treated with an endovascular approach, some of them with intracerebral AVMs for the purpose of a preoperative embolization. Emergency procedures were performed in 29 patients (78%), whereas 12 patients (32%) were treated in a delayed fashion. In 29 cases (78%) the technical outcome was reported. Complete embolization was achieved in 19 patients (51%), in 22 (59%) on first follow-up and 26 (70%) patients did not have any procedure-related complications. Out of the case-based systematic review patients, six (16%) had evidence of postprocedural brain ischemia on cranial imaging and two (5%) had fatal complications. The out of 36 reported fetal outcomes, 32 fetuses (89%) were born healthy and 4 (11%) died, either due to termination of pregnancy or maternal death.

**Table 2:** Characteristics of all cases obtained from the case-based literature review

Variable	All patients (*n* = 37)	Aneurysm (*n* = 17)	AVM ^a^ (*n* = 19)	AVF ^b^ (*n* = 1)
*Age in years (mean* ± *SD)*	27.4 ± 6	28.6 ± 5.1	26.7 ± 6.4	19
*Gestation week, median (IQR)*	21 (16–27)	27 (1.5–30)	19 (14–22)	19
*Type of bleeding*				
Intracerebral hemorrhage; *n* (%)	14 (38%)	1 (6%)	12 (63%)	1 (100%)
Subarachnoid hemorrhage, *n* (%)	21 (57%)	17 (100%)	4 (21%)	0
Intraventricular hemorrhage, *n* (%)	7 (19%)	3 (18%)	6 (32%)	0
*Initial symptoms*				
Headache; *n* (%)	27 (73%)	14 (82%)	12 (63%)	1 (100%)
Vomiting, *n* (%)	11 (30%)	4 (24%)	6 (32%)	1 (100%)
Focal symptoms, *n* (%)	12 (32%)	4 (24%)	6 (32%)	1 (100%)
Seizure, *n* (%)	3 (8%)	3 (18%)	0	0
Reduced consciousness, *n* (%)	10 (27%)	5 (29%)	5 (26%)	0
No information, *n* (%)	5 (14%)	0	5 (26%)	0
*Number of pregnancies*				
Primipara, *n* (%)	10 (27%)	5 (29%)	4 (21%)	1 (100%)
Multipara, *n* (%)	12 (32%)	5 (29%)	7 (37%)	0
No information, *n* (%)	19 (51%)	9 (53%)	9 (47%)	1 (100%)
*Elective (planned) procedure*	12 (32%)	5 (29%)	6 (32%)	1 (100%)
*Emergency procedure*	29 (78%)	14 (82%)	14 (74%)	1 (100%)
*Type of delivery*				
Vaginal, *n* (%)	8 (22%)	4 (24%)	4 (21%)	0
Cesarean, *n* (%)	23 (62%)	10 (59%)	11 (58%)	1 (100%)
Termination, *n* (%)	4 (11%)	1 (6%)	3 (16%)	0
No information, *n* (%)	5 (14%)	3 (18%)	2 (11%)	0
*Time of delivery*				
On term, *n* (%)	23 (62%)	12 (32%)	10 (53%)	1 (100%)
Preterm, *n* (%)	7 (19%)	4 (24%)	2 (11%)	1 (100%)
Termination; death of mother, *n* (%)	4 (11%)	1 (6%)	3 (16%)	0
No information, *n* (%)	7 (19%)	2 (12%)	5 (26%)	0
*Fetal outcome*				
Healthy, *n* (%)	32 (86%)	15 (88%)	15 (79%)	1 (100%)
Dead, *n* (%)	4 (11%)	1 (6%)	3 (16%)	0
No information, *n* (%)	5 (14%)	3 (18%)	2 (11%)	0
*Maternal complications*				
None, *n* (%)	26 (70%)	13 (76%)	11 (58%)	1 (100%)
Ischemic, *n* (%)	6 (16%)	3 (18%)	3 (16%)	0
Hemorrhagic, *n* (%)	0	0	0	0
Fatal, *n* (%)	2 (5%)	1 (6%)	1 (5%)	0
No information, *n* (%)	7 (19%)	2 (12%)	5 (26%)	–
*Postoperative embolization status *				
Full, *n* (%)	19 (51%)	15 (88%)	3 (16%)	1 (100%)
≥ 80%, *n* (%)	3 (8%)	0	3 (16%)	0
Partial, *n* (%)	7 (19%)	1 (6%)	6 (32%)	0
No information, *n* (%)	12 (32%)	3 (18%)	8 (42%)	1 (100%)
*First follow-up embolization status *				
Full, *n* (%)	22 (59%)	15 (88%)	6 (32%)	1 (100%)
≥ 80%, *n* (%)	3 (8%)	0	3 (16%)	0
Partial, *n* (%)	4 (11%)	1 (6%)	6 (32%)	0
No information, *n* (%)	12 (32%)	3 (18%)	8 (42%)	1 (100%)
*Hunt and Hess score*				
1, *n* (%)	–	2 (12%)	–	–
2, *n* (%)	–	4 (24%)	–	–
3, *n* (%)	–	3 (18%)	–	–
4, *n* (%)	–	1 (6%)	–	–
No information, *n* (%)	–	9 (53%)	–	–
*Spetzler-Martin grade*				
1, *n* (%)	–	–	5 (26%)	–
2, *n* (%)	–	–	4 (21%)	–
3, *n* (%)	–	–	3 (16%)	–
4, *n* (%)	–	–	3 (16%)	–
5, *n* (%)	–	–	0	–
No information, *n* (%)	–	–	5 (26%)	–
*Perioperative anticoagulation *				
Heparin, *n* (%)	–	11 (65%)	1 (5%)	1 (100%)
No information, *n* (%)	–	8 (47%)	18 (95%)	1 (100%)
*Antiplatelet treatment*				
ASA ^c^, *n* (%)	–	1 (6%)	–	–
No information, *n* (%)	–	16 (94%)	19 (100%)	1 (100%)
*Duration of anticoagulation/antiplatelet treatment*				
Stopped after procedure, *n* (%)	–	3 (18%)	–	–
< 24 h, *n* (%)	–	1 (6%)	–	–
< 7 days, *n* (%)	–	2 (12%)	–	1 (100%)
> 7 days, *n* (%)	–	1 (6%)	–	–
No information, *n* (%)	–	13 (76%)	29 (100%)	1 (100%)

*IQR* interquartile range, ^a^ *AVM* arteriovenous malformation, ^b^ *AVF* arteriovenous fistula, ^c^ *ASA* acetylsalicylic acid

Twenty-three patients (62%) were able to deliver at term and 7 (19%) had preterm delivery, consisting of 8 vaginal deliveries (22%) and 23 CS (62%).

### Calculation of Fetal Radiation Dose

Additionally, we calculated the fetal radiation dose due to a single diagnostic digital subtraction angiography (DSA) and therapeutic endovascular procedure. Details are reported in the Online Resources file.

The total dose area product (DAP) of the whole procedure (diagnostic angiography and therapeutic endovascular procedure) was 15.8 Gy cm^2^. Considering the tube voltages and the collimated field areas of the 157 irradiation events, and the estimated distance between the radiation fields and the fetus (30 cm), a conversion factor of < 0.001 mSv/Gy*cm^2^ for the calculation of the uterus dose from the DAP was selected. We used the uterus dose in mSv as an approximation for the fetal dose in mGy.

Accordingly, we calculated a fetal radiation dose estimate of 0.001 mGy/Gy cm^2^ × 15.8 Gy cm^2^ = 0.02 mGy due to scattered radiation.

## Discussion

Here, we present the current literature and a case series of pregnant patients presenting with a ruptured cerebrovascular aneurysm or AVMs who underwent endovascular treatment. The mean age of our case series was quite similar to the mean age of the literature review patients (31 ± 7 years vs. 27.5 ± 5.9 years), providing a solid initial position for further comparison.

The incidence of ruptured intracranial aneurysms in the general population varies across different regions of the world. An age-adjusted annual incidence in European and Asian countries of 2 cases per 100,000 persons has been reported by a World Health Organization study [8]. Comparatively, the pregnant population exhibits a higher annual incidence of subarachnoid hemorrhage of 10 cases per 100,000 cases [9]. The median age of patients with ruptured intracranial aneurysms is 52 years in the general population, whereas Korhonen et al. reported a mean age of 32.8 (± 7.0) years in a pregnant study population suffering from ruptured intracranial aneurysms [10]; however, the exact age of patients with pregnancy-related aneurysms remains unknown.

Data reporting the true prevalence of cerebral AVMs in the general population are sparse. Autopsy studies reported a prevalence of 5–613 cases per 100,000 persons, whereas retrospective and prospective community-based studies reported an AVM detection rate ranging from 1.11–1.34 per 100,000 person-years in unselected populations.

The mean age of patients diagnosed with cerebral AVMs ranges from 28 to 39 years. Intracranial hemorrhage is the most common presentation of AVMs in up to 50% of the patients, with a median age of 52 years at the time of rupture [11, 12]; however, there are no studies evaluating the prevalence of unruptured cerebral AVMs in the pregnant population but the incidence and risk factors of an AVM hemorrhage in the general population are well studied. Still, it remains controversial whether pregnant patients are at higher risk for AVM rupture. Liu et al. reported no increased risk of hemorrhage during pregnancy [13], whereas several studies found an increased AVM hemorrhage risk in pregnancy [1, 14, 15]. These differences may be explained by unique population attributes which in turn could impact the risk of AVM hemorrhage during pregnancy.

The younger age of pregnant patients, compared to the general population, suffering from ruptured intracranial aneurysms could be explained by the unique and physiological changes of pregnancy, which in turn contribute to vascular stress [16]. Although the exact prevalence of ruptured cerebral AVMs in the general and pregnant populations remains unknown, a higher risk of hemorrhage during pregnancy seems plausible due to increased cerebral blood flow and higher levels of vasoactive hormones [17].

The overall maternal outcome was good in both populations, our case series and literature review patients. The mRs at 90 days or at discharge was reported in 11 cases in our series and 7 of these patients (64%) made an excellent recovery without significant neurological deficits (mRS ≤ 1) at 90 days or at discharge. The literature review population was able to be discharged without any periprocedural maternal complications in 70% of the cases. Regarding the outcome after spontaneous subarachnoid hemorrhage in the general population, a recent population-based cohort study reported that a favorable outcome, defined as an mRS score of 0–2, was reached in 51.5% after 12 months, whereas the overall mortality rate was 23.7% [18]. According to the international subarachnoid aneurysm trial (ISAT), 52.9% of patients presenting with ruptured intracranial aneurysms treated with an endovascular approach recovered with no symptoms or minor symptoms (mRS ≤ 1) [19].

However, these differences may be explained by the lower mean age of our study populations, the different hemorrhage etiology or publication bias.

Regarding the techniques used, especially for aneurysm treatment, a straightforward approach should be considered for the treatment of pregnant patients. The use of double antiplatelet treatment and stents or flow diverter devices could potentially expose the patients to an increased risk of in-stent thrombosis during pregnancy and the puerperium.

### Fetal Radiation Exposure

During the past decade, the use of radiological examinations for the evaluation of neurological conditions in pregnant patients has increased [20]. Still, fetal radiation protection is of particular concern for the diagnosis, treatment strategy planning and also the treatment itself, as endovascular procedures have been increasingly considered as a treatment option in pregnancy [5]. Two-dimensional DSA entails a radiation burden of approximately 2.71 mSv [21]; however, this represents an estimated radiation dose to the mother and does not reflect the radiation dose to the fetus. Considering that the fetus is not in the directly irradiated area and shield protection is used, it is only exposed to scattered radiation. Our calculated fetal radiation dose of 0.02 mGy during diagnostic angiography and therapeutic endovascular procedure, confirmed the relatively low dose to the fetus under appropriate shielding protection of the uterus and is similar to fetal radiation doses previously calculated by other interventionalists [22, 23]. Assuming that the cumulative fetal radiation dose should never exceed 100 mGy [20], endovascular procedures can be performed safely in pregnant patients. In patients with appropriate aortic arch anatomy and in centers where alternative access is practiced regularly, using a radial approach could be a potential alternative with possible reduction of the fetal radiation exposure. In rare cases, a femoral approach could be complicated by the hemodynamic effects resulting from aortocaval compression occurring during late pregnancy and consequently prolongate the fetal radiation exposure [24]. Decreasing the beam angle and shortening the duration of fluoroscopy procedures can further minimize the fetal radiation exposure. Finally, according to the Canadian stroke best practice consensus statement, pregnancy should not be regarded as a contraindication for angiography and endovascular treatment, and a delay or deferment of necessary maternal care secondary to pregnancy is not considered reasonable [25].

### Timing and Mode of Delivery

A further important consideration is the mode and timing of delivery, whereas lacking guidelines make it even harder to determine the best approach. Therefore, a multidisciplinary discussion is required. Both, the fetal and maternal conditions, should be taken into consideration and individually approached, such as balancing the risks of premature birth and the maternal clinical state.

While a history of stroke is not an absolute contraindication to vaginal delivery, it still seems that CS is more frequently performed, with the particular aim of avoiding increased intracranial pressure, which may result in rebleeding. In fact, the odds of a cerebrovascular event following CS delivery is significantly higher compared to a vaginal delivery [25].

In our case series, the number of vaginal deliveries and CS was balanced and both routes were successfully performed with good maternal and fetal outcomes, although admittedly with potential selection bias. In contrast, CS was the delivery modality of choice in the reviewed literature. Here, too, the overall maternal and fetal outcomes were good independent of delivery type; however, although the frequency is low, the maternal risks of CS such as bleeding, organ injury, infections and thrombosis should not be dismissed. Taken together, our results are in line with current guidelines stating that a vaginal delivery in this particular patient population is at least as feasible as CS.

## Limitations

This study has several limitations. First, we present a retrospectively collected case series with a limited number of patients. Second, technical and clinical outcomes were assessed by the responsible interventionalists and therefore not blinded. Lastly, the number of cases of ruptured cerebrovascular malformations in pregnant women, especially those treated with an endovascular approach, was limited.

## Conclusion

Taken together, our literature review and case series indicate that the acute endovascular treatment of ruptured cerebrovascular pathologies, such as aneurysms and AVMs, in pregnant women is safe and feasible, regarding both, the maternal and fetal outcomes. Furthermore, vaginal delivery seems to bear a smaller risk of cerebrovascular events in pregnant patients diagnosed with brain aneurysms and AVMs.

To help build a stronger knowledge base, clinicians are urged to collect and share data regarding the management of pregnant women suffering from intracranial hemorrhage caused by cerebrovascular lesions.

## Supplementary Information

The Supplementary Information file contains the detailed information about the applied search strings for the systematic literature search and a detailed description of the fetal radiation dose calculation.
