# Supplementary material for: Endovascular Treatment and Peri-interventional Management of Ruptured Cerebrovascular Lesions During Pregnancy: Case Series and Case-based Systematic Review
Source: Clin Neuroradiol. 2023 May 31;33(3):833–42. doi: 10.1007/s00062-023-01287-x (PMC10449989; doi:10.1007/s00062-023-01287-x)
Supplement: Supplementary file 1 — The Supplementary Information file contains the detailed information about the applied search strings for the systematic literature search and a detailed description of the fetal radiation dose calculation. [file 62_2023_1287_MOESM1_ESM.pdf]

Supplementary Information

Search strings

The search strings used were as followed: (Brain OR Neuro OR Neurovascular OR Cerebral OR Cranial OR Intracranial OR intraparenchymal) AND (SAH OR SAB OR AVM OR Subarachnoid hemorrhage OR Subarachnoid bleeding OR Arteriovenous Malformations OR Aneurysm OR dAVF OR arteriovenous fistula OR arteriovenous) AND (Pregnant women OR pregnancy OR Pregnant).

Calculation of fetal radiation dose

The fetal radiation dose due to a single neuroradiological intervention was estimated as follows: The peak skin dose calculation module of a commercial dose management software (Radimetrics, Bayer Healthcare) was used to check the beam positions of the 157 individual irradiation events within the procedure. Except for 2 out of 157 irradiation events, the primary radiation beam position was in the head and neck region (the other two irradiation events were closer to the fetus but had a negligible combined DAP of 0.007 Gy cm^2). A conservative (i.e. low) estimate of the distance between the primary radiation fields and the fetus of 30 cm was obtained using a publicly available web tool (www.fetaldose.org).

The anthropomorphic phantom corresponding to a gestational age of 3-6 months was selected, as the patient was 23 weeks pregnant at the time of the procedure. The web tool was only used to get an estimate of the distances, not the fetal dose.

The total DAP of the whole procedure was 15.8 Gy cm^2. Considering the tube voltages and the collimated field areas of the 157 irradiation events, and the estimated distance between the radiation fields and the fetus (30 cm, see above), a conversion factor of <0.001 mSv/Gy*cm^2 for the calculation of the uterus dose from the DAP was selected from Table B-9 of the DGMP-Bericht Nr. 7 from 2019 (indikation/). The uterus dose (in mSv) was used as an approximation for the fetal dose (in mGy). Thus, a conservative, rough estimation of the fetal dose of 0.001 mGy/Gy cm^2 * 15.8 Gy cm^2 = 0.02 mGy due to scattered radiation was obtained.
